# Immunologic Gene Signature Analysis Correlates Myeloid Cells and M2 Macrophages with Time to Trabectedin Failure in Sarcoma Patients

**DOI:** 10.3390/cancers14051290

**Published:** 2022-03-02

**Authors:** Brett A. Schroeder, Yuzheng Zhang, Kimberly S. Smythe, Parth Desai, Anish Thomas, Pedro Viveiros, Borislav A. Alexiev, Farres Obeidin, Eleanor Y. Chen, Lee D. Cranmer, Michael J. Wagner, Robin L. Jones, Jean S. Campbell, Robert H. Pierce, Qianchuan He, Seth M. Pollack

**Affiliations:** 1 National Cancer Institute, National Institutes of Health, Bethesda, MD 20829, USA; brett.schroeder@nih.gov; 2 Clinical Research Division, Fred Hutchinson Cancer Research Center, Seattle, WA 98109, USA; yzhang@fredhutch.org (Y.Z.); ksmythe@fredhutch.org (K.S.S.); 3 Developmental Therapeutics Branch, Center for Cancer Research, National Cancer Institute, National Institutes of Health, Bethesda, MD 20829, USA; parth.desai@nih.gov (P.D.); anish.thomas@nih.gov (A.T.); 4 Department of Medicine, Northwestern University Feinberg School of Medicine, Chicago, IL 60611, USA; pedro.viveiros@northwestern.edu; 5 Department of Pathology, Northwestern University Feinberg School of Medicine, Chicago, IL 60611, USA; borislav.alexiev@northwestern.edu (B.A.A.); farres.obeidin@nm.org (F.O.); 6 Department of Laboratory Medicine and Pathology, University of Washington, Seattle, WA 98195, USA; eleanor2@uw.edu (E.Y.C.); campjs@uw.edu (J.S.C.); 7 Department of Medical Oncology, University of Washington, Seattle, WA 98195, USA; lcranmer@seattlecca.org (L.D.C.); wagnermj@uw.edu (M.J.W.); 8 Sarcoma Unit, Royal Marsden Hospital NHS Trust and Institute of Cancer Research, London SW3 6JJ, UK; robin.jones4@nhs.net; 9 Sensei Biotherapeutics, Boston, MA 02120, USA; rpierce@senseibio.com; 10 Division of Oncology, Northwestern University, Chicago, IL 60611, USA

**Keywords:** trabectedin, sarcoma, M2 macrophage, PD-L1, myeloid cells

## Abstract

**Simple Summary:**

Trabectedin is an FDA-approved chemotherapy with demonstrated benefit for some sarcoma subtypes, particularly in the metastatic setting. Although some patients receiving trabectedin have only modest benefit, other patients are exceptional responders. While several mechanisms of action have been suggested for trabectedin, we suspect that it has a role in immune modulation, and we hypothesized that the presence of specific immune cells and related gene expression patterns may help identify which patients are more likely to benefit from trabectedin therapy. We confirmed that six immunologic gene signatures are significantly associated with up to 7-year survival, notably myeloid-derived suppressor cells and M2 macrophages, using a gene set analysis tool to evaluate group associations. Furthermore, tumors characterized with this type of immunosuppressive microenvironment and high PD-L1 expression are less likely to benefit from trabectedin, which could guide providers in treatment decisions.

**Abstract:**

Patients with metastatic soft tissue sarcoma (STS) have a poor prognosis and few available systemic treatment options. Trabectedin is currently being investigated as a potential adjunct to immunotherapy as it has been previously shown to kill tumor-associated macrophages. In this retrospective study, we sought to identify biomarkers that would be relevant to trials combining trabectedin with immunotherapy. We performed a single-center retrospective study of sarcoma patients treated with trabectedin with long-term follow-up. Multiplex gene expression analysis using the NanoString platform was assessed, and an exploratory analysis using the lasso-penalized Cox regression and kernel association test for survival (MiRKAT-S) methods investigated tumor-associated immune cells and correlated their gene signatures to patient survival. In total, 147 sarcoma patients treated with trabectedin were analyzed, with a mean follow-up time of 5 years. Patients with fewer prior chemotherapy regimens were more likely to stay on trabectedin longer (pairwise correlation = −0.17, *p* = 0.04). At 5 years, increased PD-L1 expression corresponded to worse outcomes (HR = 1.87, *p* = 0.04, *q* = 0.199). Additionally, six immunologic gene signatures were associated with up to 7-year survival by MiRKAT-S, notably myeloid-derived suppressor cells (*p* = 0.023, *q* = 0.058) and M2 macrophages (*p* = 0.03, *q* = 0.058). We found that the number of chemotherapy regimens prior to trabectedin negatively correlated with the number of trabectedin cycles received, suggesting that patients may benefit from receiving trabectedin earlier in their therapy course. The correlation of trabectedin outcomes with immune cell infiltrates supports the hypothesis that trabectedin may function as an immune modulator and supports ongoing efforts to study trabectedin in combination with immunotherapy. Furthermore, tumors with an immunosuppressive microenvironment characterized by macrophage infiltration and high PD-L1 expression were less likely to benefit from trabectedin, which could guide clinicians in future treatment decisions.

## 1. Introduction

Trabectedin is an FDA-approved chemotherapy discovered through a high-throughput screen, originally isolated from the tunicate *Ecteinascida turbinata.* A multicenter Phase III trial demonstrated improved progression-free survival (PFS) with trabectedin versus dacarbazine in patients with metastatic liposarcoma and leiomyosarcoma after disease progression on conventional chemotherapy (4.2 vs. 1.5 months) [1]. A randomized trial demonstrated improved overall survival (OS) in patients with translocation-associated sarcomas when treated with trabectedin versus best supportive care [2]. Trabectedin binds to the minor groove of DNA strands, inducing apoptosis through double-strand breaks [3,4], and inhibits the binding of transcription factors to target gene promoters [5]. However, there is also evidence suggesting that some efficacy may not entirely be due to direct cytotoxic activity on tumor cells, but rather to immunomodulation of the tumor microenvironment (TME). Germano et al. demonstrated that human myxoid/round cell liposarcoma (MRCL) tumor lines treated with trabectedin have decreased levels of key inflammatory cytokines such as IL-6, CCL2, and CXCL8 [6]. The same group later demonstrated that trabectedin depletes monocytes in murine tissues and that some antitumor effect is mediated by cytotoxicity specific to mononuclear phagocytes, including tumor-associated macrophages (TAMs), within the TME [7].

Although the demonstrated PFS improvement compared to dacarbazine for patients receiving trabectedin is approximately 4 months [8], there is a minority of exceptional responders who have stable disease past 12 months [9]. Because we suspect that immune modulation may play a role in the activity of trabectedin, we hypothesized that the presence of specific immune infiltrates and immune-related gene expression might help identify patients likely to benefit from trabectedin therapy. In order to better characterize the association of immunologically relevant gene sets with clinical outcomes, we applied a method of gene set analysis to test group associations originally developed to serve the field of microbiome analysis called microbiome regression-based kernel association test with survival outcomes (MiRKAT-S) [10]. While this analysis of gene signatures cannot be used directly to predict patient response, it can be a highly useful descriptive analysis for factors important to clinical outcomes.

## 2. Materials and Methods

### 2.1. Patients and Data Collection

All data collection and analysis of tumor samples were performed under an IRB-approved retrospective study. We performed a retrospective search of the University of Washington (UW) and Fred Hutchinson Cancer Research Center (FHCRC) using the Caisis open-source, web-based data management system to identify sarcoma patients treated with TRB prior to 2016. Formalin-fixed paraffin-embedded (FFPE) tumor samples were requested in both slides and curls whenever available. Once abstracted data and tumor samples were collected, all information and samples were deidentified for subsequent analysis.

### 2.2. Multiplex Immunohistochemistry

Four-micrometer FFPE tissue sections were baked for 1 h at 60 °C, dewaxed (BOND Dewax Solution), and then stained on a Leica BOND Rx stainer. Antigen retrieval and antibody stripping were performed at 100 °C using Epitope Retrieval Solution 2 and Bond Wash Solution. All other steps were performed at room temperature. Endogenous peroxidase was blocked with 3% H_2_O_2_ for 8 min, followed by protein blocking with TCT buffer (0.05 M Tris, 0.15 M NaCl, 0.25% casein, 0.1% Tween 20, and pH 7.6) for 30 min. A high stringency wash of high-salt TBST solution (0.05 M Tris, 0.3 M NaCl, and 0.1% Tween 20, pH 7.2–7.6) was performed after the secondary and tertiary applications. The first primary antibody (position 1) was applied for 1 h, followed by the secondary antibody application for 10 min and the application of the tertiary TSA-amplification reagent (Akoya OPAL fluor) for 10 min. The primary and secondary antibodies were stripped with retrieval solution for 20 min before the process was repeated with the second primary antibody (position 2) starting with a new application of 3% H_2_O_2_. The process was repeated for all 6 positions. Antibody position, clone, and concentration are provided in Table S1.

Slides were stained with Spectral DAPI (Akoya) for 5 min, rinsed for 5 min, and cover-slipped with Prolong Gold Antifade. OPAL Polymer HRP Mouse plus Rabbit (Akoya) was used for all secondary applications. Slides were cured for 24 h at room temperature, and then representative images from each slide were acquired on an Akoya Vectra 3.0 Automated Imaging System. Images were spectrally unmixed using Akoya inForm software and exported as multi-image TIFF files and analyzed using HALO (Indica Labs). Regions of interest were defined based on the tumor cell expression and cell morphology. All slides were reviewed by an experienced pathologist, and staining efficiency was compared to a tonsil tissue control (Figure S1).

### 2.3. NanoString

Tissue sections were deparaffinized and RNA was extracted using the High Pure FFPET RNA isolation kit from Roche. Per sample, 50 ng (RNA content) from the cellular lysate in a final volume of 5 uL was mixed with a 3′ biotinylated capture probe and a 5′ reporter probe tagged with a fluorescent barcode, using the 360IO kit from NanoString. As per standard, probes and target transcripts were hybridized overnight at 65 °C for 12–16 h as per manufacturers’ recommendations. Hybridized samples were run on the NanoString nCounter station in the Fred Hutchinson Cancer Research Center Genomics core facility, using their high sensitivity protocol where excess capture and reporter probes were removed and transcript-specific ternary complexes were immobilized on a streptavidin-coated cartridge. The samples were scanned at maximum scan resolution capabilities using the nCounter digital analyzer [11,12].

NanoString data were normalized by samplewise mean centering, using the geometric mean of 6 spike-in control genes first and then centering the geometric mean of the 20 housekeeping genes. Log10 transformation was conducted to yield more normal distributions for gene expressions. Cox regression analysis was used to find the association between NanoString expressions and patient outcome, including (A) the time to trabectedin failure after adjusting for tumor grade and (B) up to 5-year OS after adjusting for the number of treatments and tumor grade. Based on the 53 genes that showed significant association (*p* < 0.05) from the marginal Cox regression in (A), we then applied a lasso-penalized Cox model to select a combination panel to best predict the time to drug failure. Eleven genes were selected by lasso as the best predictive combination.

Gene set analysis was conducted to test the association between nine cancer-related gene sets and overall survival using the MiRKAT-S package [10]. It is a kernel association test for up to 7-year overall survival after adjustment for number of drug treatments and tumor grade. Euclidean distance was used for the dissimilarity measures as kernel estimation.

To find genes correlated with time to trabectedin failure, NanoString expression was analyzed using pairwise Pearson correlation [13]. Correlation coefficient, *p*-value, and Benjamini–Hochberg adjusted p-value (also referred to as *q*-value) were outputs after adjusting for tumor grade. Cox regression was applied to TMA data for prediction at 5-year and 15-year OS. The model used adjusted covariates including number of treatments and tumor grade. All statistical analyses were conducted using the R analysis software (version 4.1.1).

## 3. Results

### 3.1. Patient Demographics

In total, we identified 147 sarcoma patients (Table 1) treated with trabectedin for our initial analysis, with a mean follow-up time of 5 years. Since this was prior to FDA approval, patients were treated on either the trabectedin expanded program (NCT01427582), or the registration trial (NCT01343277). The median age was 56 years (range 22–80), and the sample included 58 men (39.5%) and 89 women (60.5%). Thirty-two different STS histology types were documented (Table S2). The most common STS subtypes were undifferentiated pleomorphic sarcoma (UPS, 17%), nonuterine leiomyosarcoma (LMS) (16.3%), well-/dedifferentiated liposarcoma (WDLP/DDLP) (9.5%), synovial sarcoma (SS, 15%), and uterine LMS (7.5%). High-grade disease was noted in 68.7% (N = 101) of patients, with all patients having unresectable or metastatic disease.

From this cohort, 30 (21.8%) had sufficient available FFPE tissue; each was included in the TMA for mIHC and NanoString gene expression. All patients included in the TMA had either nonuterine LMS (43.3%), uterine LMS (10%), WDLP/DDLP (16.7%), myxoid liposarcoma (MRCL, 13.3%), or SS (16.7%). Twenty-three patients had only pretreatment tissue available, two patients had only post-treatment tissue available, and five patients had both pre- and post-treatment tissue included in the TMA. Patients received a mean of 6.4 cycles of trabectedin (SD = 6.1) and had a mean of 1.9 prior lines of therapy (SD = 1.4). Demographic information about patients with TMA and NanoString data is presented in Table S3.

### 3.2. Impact of Prior Treatment and Trabectedin Duration on Outcomes

**Figure 1:**
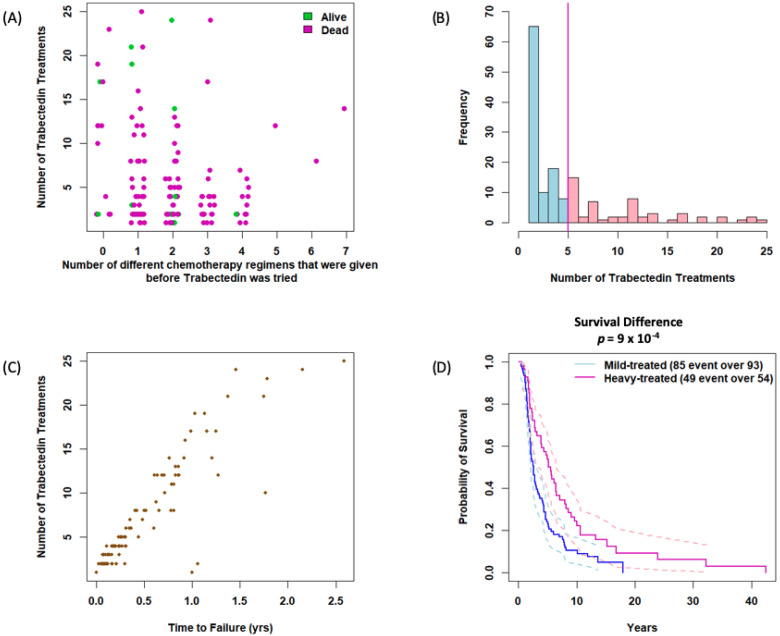
(**A**) Number of prior chemotherapy regimens vs. number of trabectedin treatments. (**B**) Number of patients with number of trabectedin treatments. (**C**) Number of trabectedin treatments until time of failure. (**D**) Survival in relation to number of trabectedin treatments in which mild-treated means fewer than 5 treatments while heavy-treated means 5 or more treatments with trabectedin.

Most patients had broad exposures to antineoplastic therapy, with a mean of 1.9 prior chemotherapy regimens before trabectedin (range 0–7 regimens). Patients with fewer prior chemotherapy regimens were more likely to continue on trabectedin longer (pairwise correlation = −0.17, *p* = 0.04) (Figure 1A). Overall, patients received an average of 5.6 trabectedin doses (range 1–25 doses). We defined “heavily trabectedin treated” as patients receiving five or more trabectedin treatments (Figure 1B) as this meant they had been on therapy beyond 3 months, and likely beyond their second set of follow-up imaging. As might be expected, the number of trabectedin treatments was directly related to time to treatment failure (Figure 1C), and heavily treated trabectedin patients showed significantly longer OS (*p* = 0.001) (Figure 1D). While only 23% of patients who received fewer than five cycles of trabectedin were alive at 5 years (95% CI: 0.15, 0.32), 54% of those who received five or more cycles were alive at 5 years (95% CI: 0.4, 0.66) (Table S3).

We performed similar analyses for patients with specific STS subtypes. While not all STS subtypes had a sufficient number of patients to draw conclusions, for nonuterine LMS patients we found a trend toward longer OS (*p* = 0.01) in heavily treated patients (Figure S2). No statistical difference was found in relation to prior number of regimens or number of trabectedin treatments received on overall survival for synovial sarcoma or UPS/spindle cell sarcoma (Figures S3 and S4).

### 3.3. Impact of PD-L1 and Immune Infiltration on Clinical Outcome

**Figure 2:**
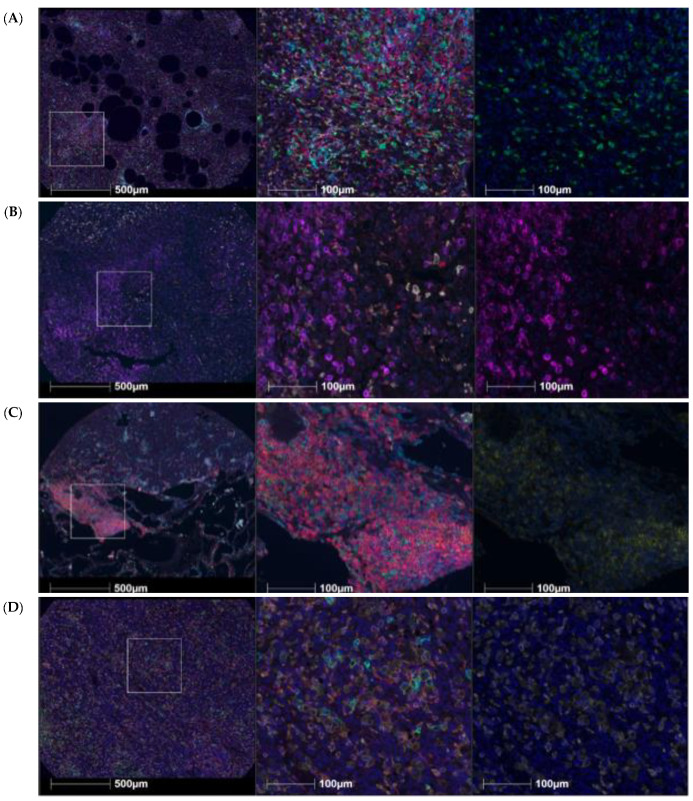
Tissue microarrays for sarcoma subtypes. CD8 (green), CD4 (red), PD-1 (yellow), PD-L1 (magenta), HLA-DR (cyan), CD68/CD163 (white), DAPI (blue). (**A**) Liposarcoma mIHC with high CD8 expression (green, far right). (**B**) Nonuterine leiomyosarcoma with high PD-L1 expression (magenta, far right). (**C**) Nonuterine leiomyosarcoma with high PD-1 expression (yellow, far right). (**D**) Synovial sarcoma with high PD-1 expression (yellow, far right).

We analyzed T cell and macrophage infiltration using a panel staining for CD4, CD8, CD68/CD163, PD1, PD-L1, and HLA-DR in pretreatment samples (Figure 2). We observed dense regions of CD8-expressing cells within liposarcoma specimens (Figure 2A). Consistent with our prior findings [14], PD1 levels in leiomyosarcoma were nearly 4× greater than those in liposarcoma and 8× greater than those in synovial sarcoma. The highest expression of both PD-L1 and PD1 was seen in nonuterine LMS samples (Figure 2B,C). PD-L1 levels in nonuterine LMS were 60× higher than those in liposarcoma and 240× greater than those in synovial sarcoma. When evaluating synovial sarcoma samples, the most prevalent immune marker was CD68/CD163 (Figure 2D). Although only 4% of total synovial sarcoma cells were immune cells, over 85% of those had positive macrophage markers.

**Figure 3:**
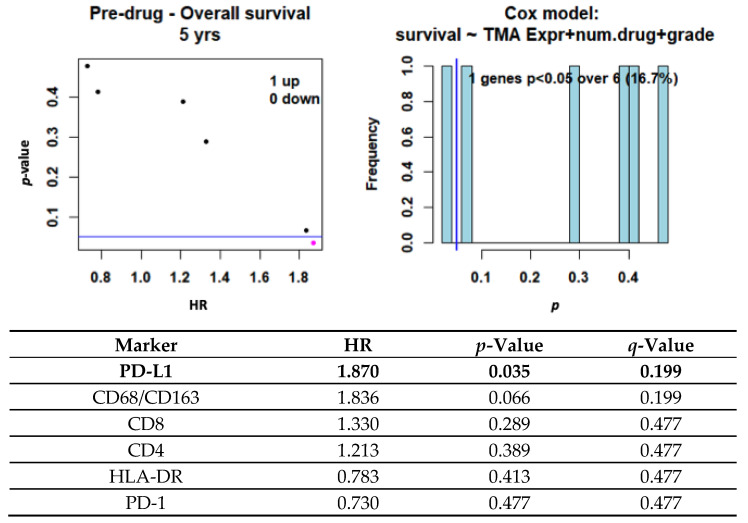
Overall survival. Pretreatment TMA samples quantified with mIHC (N = 28). Cox model controlled for tumor grade and number of drug treatments showing markers predictive of overall survival at 5 years.

Using a Cox regression model, we performed univariate analysis investigating whether any pretreatment TMA staining pattern was associated with overall survival. Importantly, after 5 years of follow-up, increased PD-L1 expression corresponded to worse outcomes (HR = 1.87, *p* = 0.035, *q* = 0.199), as did macrophage infiltration (HR = 1.84, *p* = 0.066, *q* = 0.199) (Figure 3). Pearson pairwise correlations were performed for each cell marker after adjusting for tumor grade, but no single marker showed statistical significance in relation to time to trabectedin failure (Figure S5).

### 3.4. NanoString Data

**Figure 4:**
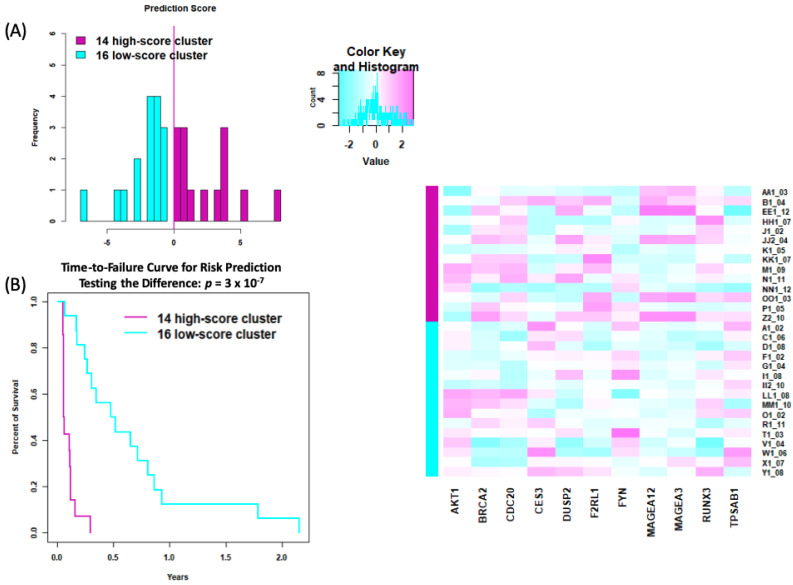
NanoString expression. (**A**) Lasso-penalized Cox model of gene expression clustering from pretreated samples in relation to time to trabectedin failure. The prediction score was calculated by the lasso coefficient multiplied by the lasso-selected gene expressions, where a high cluster score is >0 and a low cluster score is <0. (**B**) Time to trabectedin treatment failure is based on high vs. low cluster scores.

We analyzed gene expression of 750 genes from the NanoString 360IO assay using a Cox regression analysis on samples prior to trabectedin treatment. Of the genes analyzed, 53 were associated with time to trabectedin treatment failure, 28 genes with a positive association and 25 genes with a negative association (Figure S6). As demonstrated by others, we found using Pearson correlation test that SS and MRCL highly express cancer–testis (CT) antigens [15,16], specifically *MAGEA3/A6* and *MAGEA12*. Further, low expression of both MAGE genes demonstrated favorable trabectedin treatment outcomes (correlation = −0.54, *p* = 0.003, *q* = 0.98; correlation = −0.52, *p* = 0.003, *q* = 0.98, respectively). High expression levels of *AKT1* (*p* = 0.01), *IFNGR1* (*p* = 0.01), and *BRCA2* (*p* = 0.02) were predictive of early trabectedin treatment failure, irrespective of tumor grade. Lasso regression was used to further improve the interpretability of the marginal Cox model. Specifically, we looked to see from pretreated samples (N = 30) whether or not gene expression patterns might correlate to trabectedin response and, subsequently, to patient outcomes. The Cox lasso yielded 11 genes along with their regression coefficients. Applying these coefficients to the 11 genes of the original 30 samples, we obtained the predicted survival scores; then, based on these scores, we dichotomized the 30 samples into high-score group versus low-score group. We found that 11 of the previously mentioned 53 genes clustered to predict patient survival beyond 4 months (*p* < 0.001) (Figure 4).

### 3.5. Exploratory Immunologic Analysis Using MiRKAT-S

Pre-trabectedin expression data were analyzed with microbiome regression-based kernel association test with survival outcomes (MiRKAT-S), a software package to test whether differential compositions have an impact on censored survival outcomes at the community level. We examined whether gene signatures of immune cell function were associated with time to trabectedin failure. We analyzed gene signature patterns involved with T cell receptor (TCR) signaling, M1 and M2 macrophage activation, CD8+ T cell activation, regulatory T cells, and genes critical in myeloid-derived suppressor cell activity. Importantly, six gene signatures were statistically significant in association with up to 7-year overall survival (Table 2).

**Figure 5:**
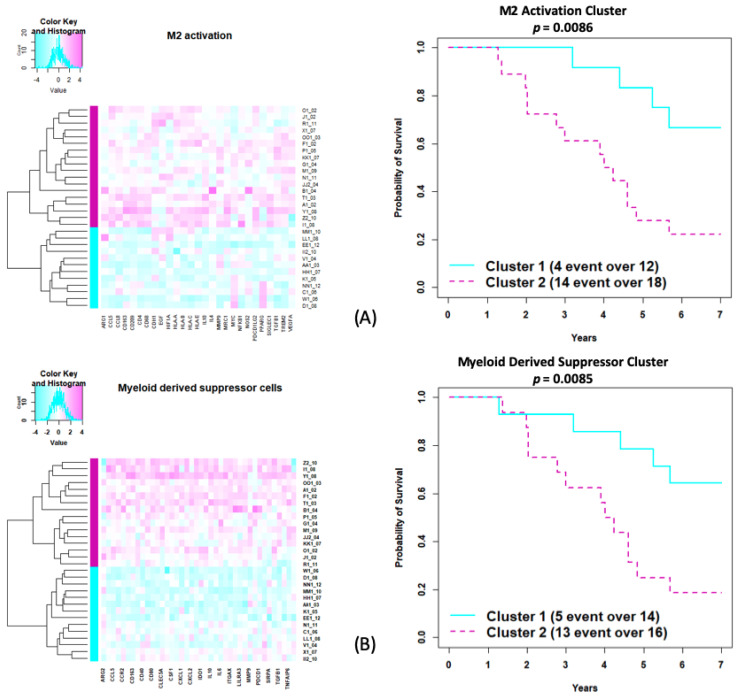
Gene set analysis using MiRKAT-S for cancer-related genes. (**A**) Genes involved in M2 macrophage activation related to survival in years (**B**). Gene cluster expression of myeloid-derived suppressor cells related to overall survival in years, with log-rank test *p*-value testing the cluster survival difference.

High expression levels of M2 macrophages and myeloid-derived suppressor cells correspond to a higher risk of death (log-rank *p* = 0.009 for both gene sets) (Figure 5).

## 4. Discussion

In our retrospective study, we found that the number of prior regimens was negatively correlated with the number of trabectedin cycles that patients received, suggesting that prior treatments may decrease trabectedin tolerability or diminish tumor sensitivity. Importantly, less than one-fourth of patients who received fewer than five cycles of trabectedin were alive at 5 years, yet more than one-half of patients who received at least five cycles of trabectedin were alive at 5 years (Table S3). Similarly, a retrospective study of 181 patients with advanced sarcoma treated with trabectedin and median follow-up of 6 years found that patients with partial response (PR) or stable disease (SD) had a better PFS (median 5.3 vs. 10.5 months, *p* = 0.001) and OS (median 13.9 vs. 33.4 months *p* = 0.01) versus patients who stopped after six cycles [8].

Trabectedin is selectively cytotoxic in vitro to human monocytes and inhibits cytokines relevant to the tumor milieu [6,17]. Likewise, others have demonstrated that trabectedin induces apoptosis not only in human primary leukemic cells, but also in selected myeloid and lymphoid immunosuppressive cells [18]. These data suggest that trabectedin behaves as an immunomodulatory drug capable of perturbing the protumor microenvironment. 

## 5. Conclusions

Here we show the importance of several key cell populations and gene signatures in relation to patient outcomes. Similar to previous findings [19], activation of M2 macrophages was negatively correlated to overall survival (Figure 5). As demonstrated previously, higher PD1/PD-L1 correlated with patient outcomes [20]. Conley et al. [21] showed that MAGE-A3 mRNA and protein expression is associated with worse OS in undifferentiated pleomorphic sarcoma/myxofibrosarcoma, which is consistent with our findings, and could make this a clinically relevant target for future investigation.

We applied MiRKAT-S to test the association between gene clusters and patient outcomes. However, there are important precautions for our study. First, due to the small sample size, the groupwise testing might not be robust enough or reliable when applied to other independent datasets for validation. Second, patients that completed additional treatment may be self-selective with fewer than the typical number of comorbidities. While these data are unlikely to help clinicians determine which patients will respond to trabectedin, they highlight biologically important associations that may be relevant to ongoing clinical trials combining trabectedin with immunotherapy. These retrospective data warrant further evaluation, and application of this model to an independent dataset and/or a future prospective study is needed.

## Figures and Tables

**Table 1:** Patient demographics and pathology with trabectedin treatment with chi-square test *p*-value evaluating the categorical distribution between mild-treated and heavy-treated clusters.

	Mild-Treated (N = 93)	Heavy-Treated (N = 54)	Total (N = 147)	*p*-Value
Age				0.35
20–30	8 (8.6%)	4 (7.4%)	12 (8.2%)	
30–40	5 (5.4%)	6 (11.1%)	11 (7.5%)	
40–50	16 (17.2%)	6 (11.1%)	22 (15.0%)	
50–60	33 (35.5%)	17 (31.5%)	50 (34.0%)	
60–70	21 (22.6%)	14 (25.9%)	35 (23.8%)	
70–80	6 (6.5%)	7 (13.0%)	13 (8.8%)	
80–90	4 (4.3%)	0 (0.0%)	4 (2.7%)	
Gender				0.027
Female	50 (53.8%)	39 (72.2%)	89 (60.5%)	
Male	43 (46.2%)	15 (27.8%)	58 (39.5%)	
Tumor Grade				0.047
Unknown	3 (3.2%)	7 (13.0%)	10 (6.8%)	
High	70 (75.3%)	31 (57.4%)	101 (68.7%)	
Intermediate	15 (16.1%)	10 (18.5%)	25 (17.0%)	
Low	5 (5.4%)	6 (11.1%)	11 (7.5%)	
Num Trabectedin Treatments				<0.001
Mean (SD)	2.323 (0.980)	11.296 (5.732)	5.619 (5.601)	
Range	1–4	5–25	1–25	
Num Previous Chemo Regimens				0.21
Mean (SD)	2.032 (1.137)	1.759 (1.466)	1.932 (1.270)	
Range	0–4	0–7	0–7	
Vital Status				0.892
Alive	8 (8.6%)	5 (9.3%)	13 (8.8%)	
Death	85 (91.4%)	49 (90.7%)	134 (91.2%)	
Follow-Up Time (years)				<0.001
N-Miss	1	0	1	
				
Mean (SD)	3.826 (3.405)	7.249 (7.621)	5.092 (5.591)	
Range	0.47–17.95	0.54–42.46	0.47–42.46	
Trabectedin Time to Failure (years)				<0.001
Mean (SD)	0.116 (0.157)	0.798 (0.512)	0.366 (0.469)	
Range	0–1.06	0.23–2.59	0–2.59	
Pathology				0.344
Leiomyosarcoma, Nonuterine	14 (15.5%)	10 (18.5%)	24 (16.3%)	
Leiomyosarcoma, Uterine	9 (9.7%)	2 (3.7%)	11 (7.5%)	
Myxoid/Round Cell Liposarcoma	2 (2.2%)	4 (7.4%)	6 (4.1%)	
Synovial Sarcoma	16 (17.2%)	6 (11.1%)	22 (15.0%)	
UPS/Spindle Cell Sarcoma	17 (18.3%)	8 (14.8%)	25 (17.0%)	
Other	35 (37.6%)	26 (48.1%)	59 (40.1%)	

**Table 2:** Gene sets related to immune system and cell activation in association with up to 7-year overall survival, with MiRKAT-S testing *p*-value and Benjamini–Hochberg adjusted *p*-value (*q*-value).

Name	Number of Genes from Original	Number of Genes in Data	Genes in Data	*p*-Value	*q*-Value
Merck 18-gene	18	17	CCL5, CD27, CD274, CD276, CMKLR1, CXCL9, CXCR6, HLA-DQA1, HLA-DRB1, HLA-E, IDO1, LAG3, NKG7, PDCD1LG2, PSMB10, STAT1, TIGIT	**0.041**	0.058
6-gene IFNg	6	6	CXCL10, CXCL9, HLA-DRA, IDO1, IFNG, STAT1	0.061	0.061
10-gene IFNg	10	10	CCR5, CXCL10, CXCL11, CXCL9, GZMA, HLA-DRA, IDO1, IFNG, PRF1, STAT1	**0.048**	0.058
TCR signaling	12	9	CCL5, CD27, CD3D, CD3G, CD4, CD74, IL2, LCK, TIGIT	**0.048**	0.058
M1 activation	28	24	CCL19, CCL5, CCL8, CD38, CD40, CXCL10, CXCL11, CXCL13, CXCL9, HLA-DPB1, HLA-DQA1, HLA-DRA, HLA-DRB1, IDO1, IFNG, IL2RA, IL6, LAG3, LILRA3, RSAD2, SIGLEC1, STAT1, TNF, TNFAIP6	**0.039**	0.058
M2 activation	35	27	ARG1, CCL5, CCL8, CD163, CD209, CD4, CD68, CDH1, EGF, HIF1A, HLA-A, HLA-B, HLA-C, HLA-E, IL10, IL4, MMP9, MRC1, MYC, NFKB1, NOS2, PDCD1LG2, PPARG, SIGLEC1, TGFB1, TREM2, VEGFA	**0.03**	0.058
T cell activation CD8	48	35	CCL5, CD2, CD247, CD27, CD3D, CD3E, CD3G, CD6, CD69, CD7, CD8B, CD96, DPP4, GNLY GZMA, GZMB, GZMK, ICOS, IL7R, IRF8, KLRB1, KLRD1, KLRK1, LAG3, LCK, LTB, LY9, NKG7, PDCD1, PDCD1LG2, PRF1, PVRIG, SH2D1A, TRAT1, ZAP70	0.052	0.058
Regulatory T cells	33	23	CD2, CD247, CD27, CD28, CD3D, CD3E, CD3G, CD4, CD5, CD6, CD70, CD96, CTLA4, DPP4, FOXP3, ICOS, IL2RA, IL2RB, LCK, LTB, SH2D1A, TRAT1, ZAP70	0.051	0.058
Myeloid-derived suppressor cells	55	40	ARG2, BTLA, CCL5, CCL8, CCR2, CD14, CD163, CD274, CD40, CD44, CD80, CD86, CLEC5A, CLEC7A, CSF1, CSF1R, CXCL1, CXCL10, CXCL2, HLA-DPB1, IDO1, IFNG, IL10, IL10RA, IL6, ITGAM, ITGAX, LILRA1, LILRA3, LILRA5, MMP9, NOS2, PDCD1, S100A8, SIRPA, STAT1, TGFB1, TGFBR2, TNFAIP6, VEGFA	**0.023**	0.058

Bold to show statistical significance.

## Data Availability

Data are available on reasonable request. All data relevant to the study are included in the article or uploaded as supplementary information.

## Supplementary Materials

The following supporting information can be downloaded at: https://www.mdpi.com/article/10.3390/cancers14051290/s1, Table S1: mIHC antibodies; Table S2: Pathology distribution by subtype, with Chi-square testing p value to test the distribution difference between drug mild-treated and drug heavy-treated groups; Table S3: TMA and NanoString demographics; Table S4: Survival summary in relation to mild-treatment (defined as <5 treatments of trabectedin), and heavy-treatment (defined as = />5 treatments of trabectedin) with columns of follow-up years; Figure S1: Tissue microarray tonsil control; Figure S2: For Non-Uterine Leiomyosarcoma subjects (N = 24); Figure S3: Synovial Sarcoma subjects (N = 22); Figure S4: UPS/Spindle Cell Sarcoma subjects (N = 25); Figure S5: TMA expression versus time to trabectedin failure adjusted for tumor grade using Pearson Pairwise Correlation, correlation testing p values, and Benjamini-Hochberg adjustment (*q*-value) (Number of subjects N = 28); Figure S6: Cox regression to analyze pre-trabectedin treatment samples demonstrating that 53 genes expres-sion data associated to time to trabectedin treatment failure.
