# Supplementary material for: Immunologic Gene Signature Analysis Correlates Myeloid Cells and M2 Macrophages with Time to Trabectedin Failure in Sarcoma Patients
Source: Cancers (Basel). 2022 Mar 2;14(5):1290. doi: 10.3390/cancers14051290 (PMC8909887; doi:10.3390/cancers14051290)
Supplement: Supplementary file 1 [file cancers-14-01290-s001.zip › cancers-1579404-supplementary.pdf]

cancers

MDPI

Article

Immunologic Gene Signature Analysis Correlates Myeloid Cells and M2 Macrophages with Time to Trabectedin Failure in Sarcoma Patients

Brett A. Schroeder, Yuzheng Zhang, Kimberly S. Smythe, Parth Desai, Anish Thomas, Pedro Viveiros, Borislav A. Alexiev, Farres Obeidin, Eleanor Y. Chen, Lee D. Cranmer, Michael J. Wagner, Robin L. Jones, Jean S. Campbell, Robert H. Pierc, Qianchuan He and Seth M. Pollack

Table S1. mIHC antibody, clone, manufacturer and concentration.

			**Panel**			
**Position**	**Antibody**	**Clone / Host**	**Manufacturer / Catalog #**	**Concentration**	**Secondary***	**OPAL Dye**
1	CD8	144B / Mouse	DAKO / M7103	0.05 µg/ml	Opal Polymer HRP	690
2	CD163	EP324 / Rabbit	Bio SB / BSB 3276	0.125 µg/ml	Opal Polymer HRP	650
	CD68	PG-M1/ Mouse	DAKO / M0876	0.038 µg/ml		
3	PD-1	D4W2J / Rabbit	Cell Signaling / 86163	0.125 µg/ml	Opal Polymer HRP	620
4	PD-L1	E1L3N / Rabbit	Cell Signaling / 13684	2.2 µg/ml	Opal Polymer HRP	540
5	HLA-DR	EP96 / Rabbit	Bio SB / BSB 6797	0.13 µg/ml	Opal Polymer HRP	570
6	CD4	EP204 / Rabbit	Epitomics / AC0173A	0.08 µg/ml	Opal Polymer HRP	520
* Secondary	Opal Polymer HRP Ms Plus Rb		Akoya / ARH1001EA	RTU		

Table S2. Pathology distribution by subtype, with Chi-square testing p value to test the distribution difference between drug mild-treated and drug heavy-treated groups.

	Mild-Treated (*N* = 93)	Heavy Treated (*N* = 54)	Total (*N* = 147)	*p* Value
Pathology				0.344
Adenosarcoma	2 (2.2%)	3 (5.6%)	5 (3.4%)	
Alveolar Rhabdomyosarcoma	0 (0.0%)	1 (1.9%)	1 (0.7%)	
Alveolar Soft Part Sarcoma	0 (0.0%)	1 (1.9%)	1 (0.7%)	
Angiosarcoma	1 (1.1%)	0 (0.0%)	1 (0.7%)	
Carcinosarcoma	1 (1.1%)	1 (1.9%)	2 (1.4%)	
Carcinosarcoma (sarcomatoid)	1 (1.1%)	0 (0.0%)	1 (0.7%)	
Clear Cell Sarcoma	2 (2.2%)	3 (5.6%)	5 (3.4%)	
Desmoplastic Round Cell Tumor	0 (0.0%)	1 (1.9%)	1 (0.7%)	
Endometrial Stromal Sarcoma	1 (1.1%)	2 (3.7%)	3 (2.0%)	
Endometrial Stromal Sarcoma	0 (0.0%)	1 (1.9%)	1 (0.7%)	
Epithelioid Sarcoma	0 (0.0%)	1 (1.9%)	1 (0.7%)	
Ewing's Sarcoma	1 (1.1%)	0 (0.0%)	1 (0.7%)	
Fibromxyosarcoma	1 (1.1%)	0 (0.0%)	1 (0.7%)	
Fibromyxoid	0 (0.0%)	1 (1.9%)	1 (0.7%)	
Fibrosarcoma	2 (2.2%)	0 (0.0%)	2 (1.4%)	
Hemangioendothelioma	1 (1.1%)	0 (0.0%)	1 (0.7%)	
Hemangiopericytoma	1 (1.1%)	0 (0.0%)	1 (0.7%)	
Intimal Sarcoma	2 (2.2%)	0 (0.0%)	2 (1.4%)	
Leiomyosarcoma	14 (15.1%)	10 (18.5%)	24 (16.3%)	
Leiomyosarcoma, Uterine	9 (9.7%)	2 (3.7%)	11 (7.5%)	
Liposarcoma	10 (10.8%)	4 (7.4%)	14 (9.5%)	
Myxofibrosarcoma	3 (3.3%)	1 (1.9%)	4 (2.7%)	
Myxoid/Round Cell Liposarcoma	2 (2.2%)	4 (7.4%)	6 (4.1%)	
Osteosarcoma	2 (2.2%)	0 (0.0%)	2 (1.4%)	
Pleomorphic Rhabdomyosarcoma	1 (1.1%)	0 (0.0%)	1 (0.7%)	
Rhabdomyosarcoma	2 (2.2%)	1 (1.9%)	3 (2.0%)	
Sarcoma	0 (0.0%)	1 (1.9%)	1 (0.7%)	
Solitary Fibrous Tumor	0 (0.0%)	2 (3.7%)	2 (1.4%)	
Synovial Sarcoma	16 (17.2%)	6 (11.1%)	22 (15.0%)	
Undifferentiated Round Cell	1 (1.1%)	0 (0.0%)	1 (0.7%)	
UPS/Spindle	17 (18.3%)	8 (14.8%)	25 (17.0%)	

Table S3. TMA and NanoString demographics.

		**Overall (*N* = 32)**
Age	20–30	1 (3.1%)
	30–40	2 (6.2%)
	40–50	7 (21.9%)
	50–60	9 (28.1%)
	60–70	7 (21.9%)
	70–80	4 (12.5%)
	80–90	2 (6.2%)
Gender	Female	18 (56.2%)
	Male	14 (43.8%)
Histology	Leiomyosarcoma	15 (46.9%)
	Leiomyosarcoma, Uterine	3 (9.4%)
	Liposarcoma	5 (15.6%)
	Myxoid Liposarcoma	4 (12.5%)
	Synovial Sarcoma	5 (15.6%)
Tumor Grade	High	21 (65.6%)
	Intermediate	8 (25.0%)
	Low	2 (6.2%)
	Unknown	1 (3.1%)
Trabectedin Treatments	Mean (SD)	6.812 (6.837)
	Range	2.000–25.000
Prior Chemotherapy Regimens	Mean (SD)	1.844 (1.347)
	Range	0.000–7.000
Overall Survival	Alive	3 (9.4%)
	Death	29 (90.6%)
Follow-up (years)	Mean (SD)	7.869 (6.669)
	Range	1.278–32.246
Time to Failure (years)	Mean (SD)	0.489 (0.637)
	Range	0.055–2.585

Table S4. Survival summary in relation to mild-treatment (defined as < 5 treatments of trabectedin), and heavy-treatment (defined as = /> 5 treatments of trabectedin) with columns of follow-up years, the number at risk, and the percentage of survival (95% CI) at each group.

Time	Num at Risk Mild-Treat	Survival Mild-Treat	95% CI Mild-Treat	Num at Risk Heavy-Treat	Survival Heavy-Treat	95% CI Heavy-Treat
0	92	0.99	(0.93,1)	54	0.98	(0.88,1)
1	87	0.93	(0.86,0.97)	53	0.96	(0.86,0.99)
2	59	0.64	(0.53,0.73)	44	0.8	(0.66,0.88)
**3**	**38**	**0.41**	**(0.31,0.51)**	**37**	**0.67**	**(0.52,0.78)**
**4**	**32**	**0.34**	**(0.25,0.44)**	**33**	**0.59**	**(0.45,0.71)**
**5**	**21**	**0.23**	**(0.15,0.32)**	**30**	**0.54**	**(0.4,0.66)**
**6**	**16**	**0.18**	**(0.11,0.27)**	**23**	**0.43**	**(0.29,0.55)**
7	15	0.17	(0.1,0.25)	18	0.34	(0.22,0.47)
8	10	0.12	(0.06,0.2)	17	0.32	(0.2,0.45)
9	9	0.1	(0.05,0.18)	14	0.26	(0.15,0.39)
10	7	0.09	(0.04,0.16)	12	0.22	(0.12,0.34)

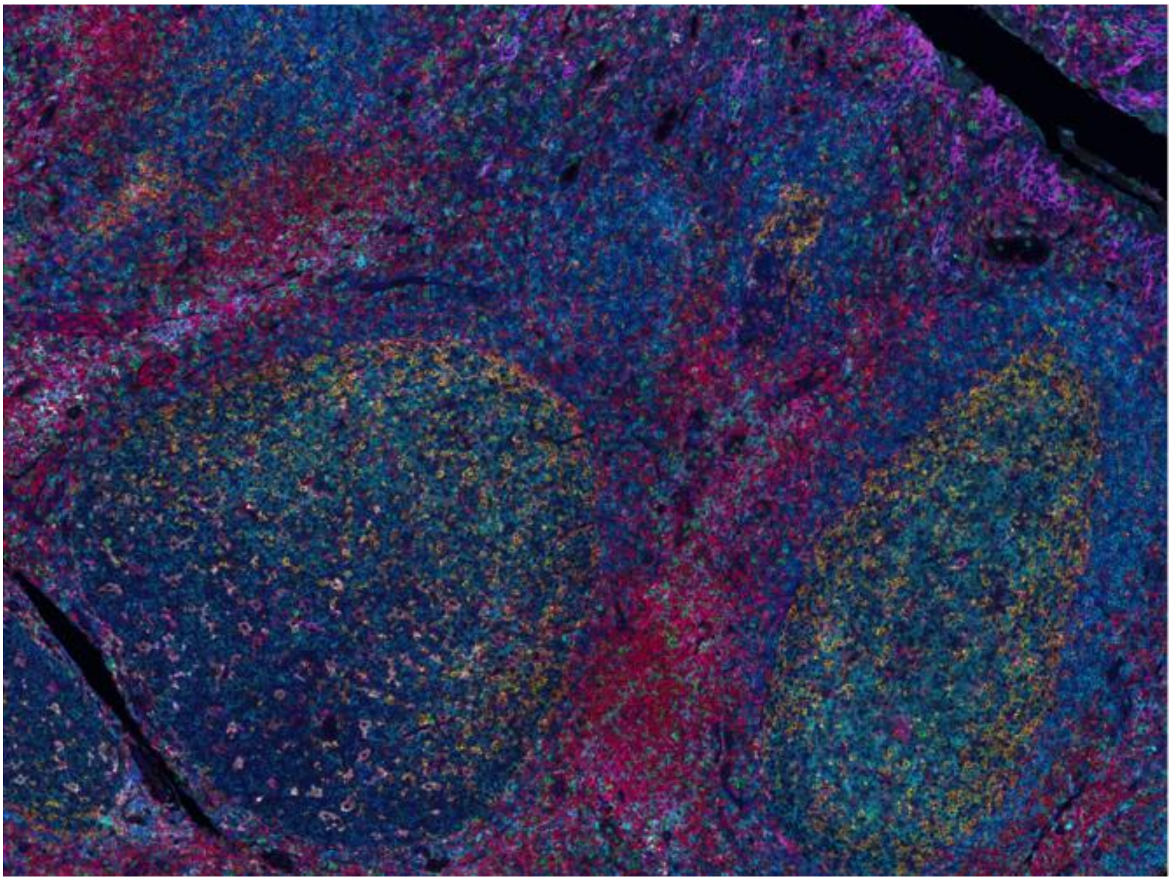

Figure S1. Tissue microarray tonsil control: CD8 (green), CD4 (red), PD-1 (yellow), PD-L1 (magenta), HLA-DR (cyan) macrophage (white), DAPI (blue).

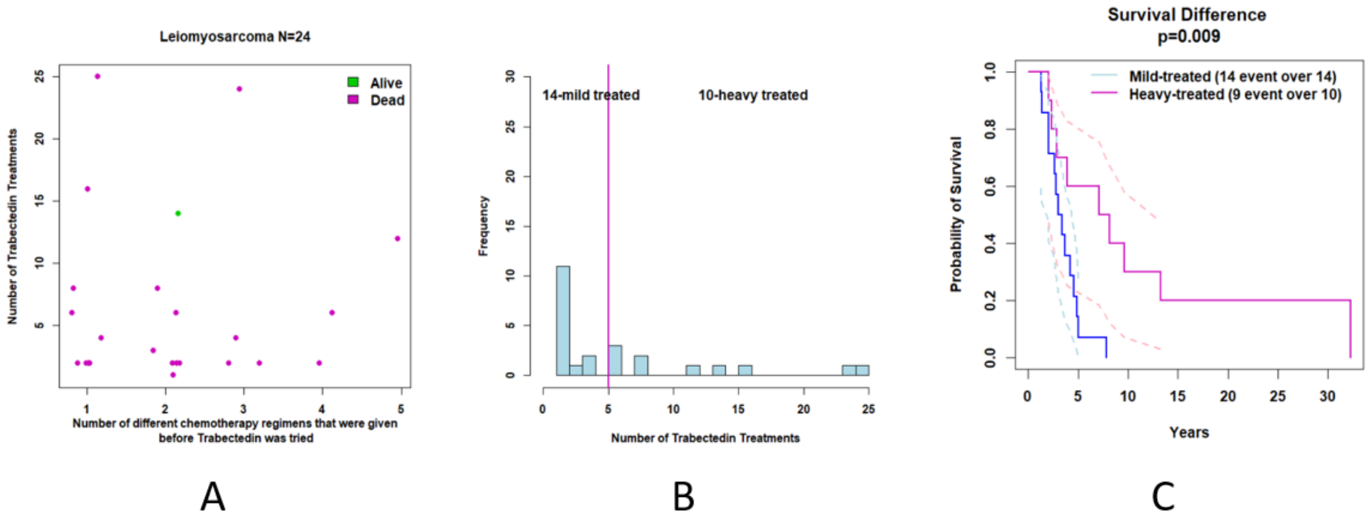

Figure S2. For Non-Uterine Leiomyosarcoma subjects (*N* = 24). (**A**) Number of chemotherapy regimens prior to trabectedin therapy, no significant correlation (Pairwise correlation = 0.022, correlation test p = 0.92). (**B**) Overall survival in relation to number of trabectedin treatments received, where heavily treated means greater than or equal to five. There is significant difference between mild-treated and heavy-treated groups with log rank test p value = 0.009. (**C**) Overall survival in relation to number of prior chemo treatments where less-chemo-treated means less than five prior chemotherapy regimens, and more-chemo-treated means greater or equal to three prior chemotherapy regimens.

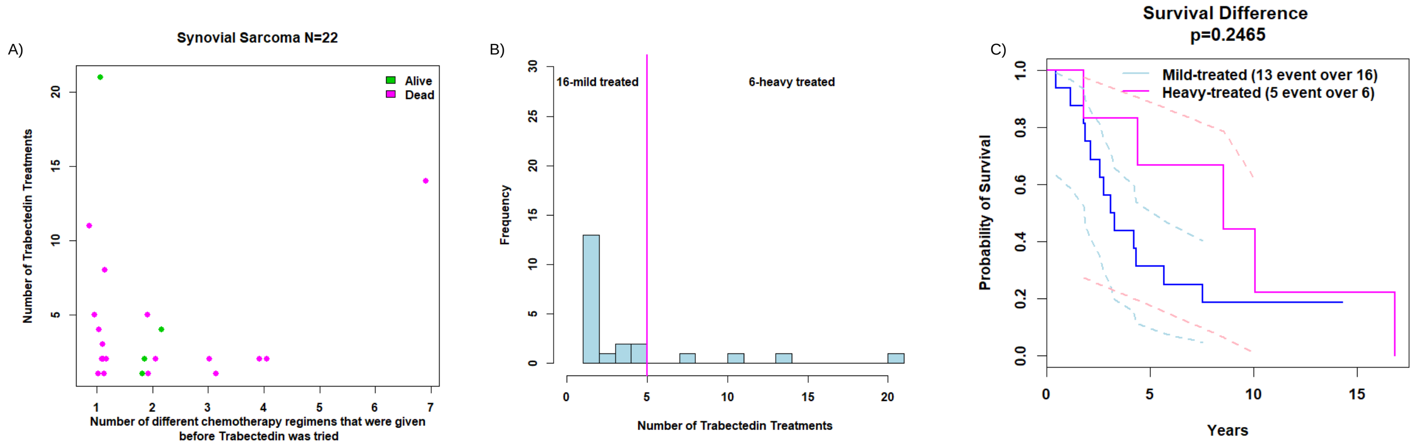

Figure S3. Synovial Sarcoma subjects (N = 22). (**A**) Number of chemotherapy regimens prior to trabectedin therapy. (**B**) Number of patients with number of trabectedin treatments received. (**C**) Survival in relation to number of trabectedin treatments received, where heavy treated means greater than or equal to five. No significant survival difference between groups.

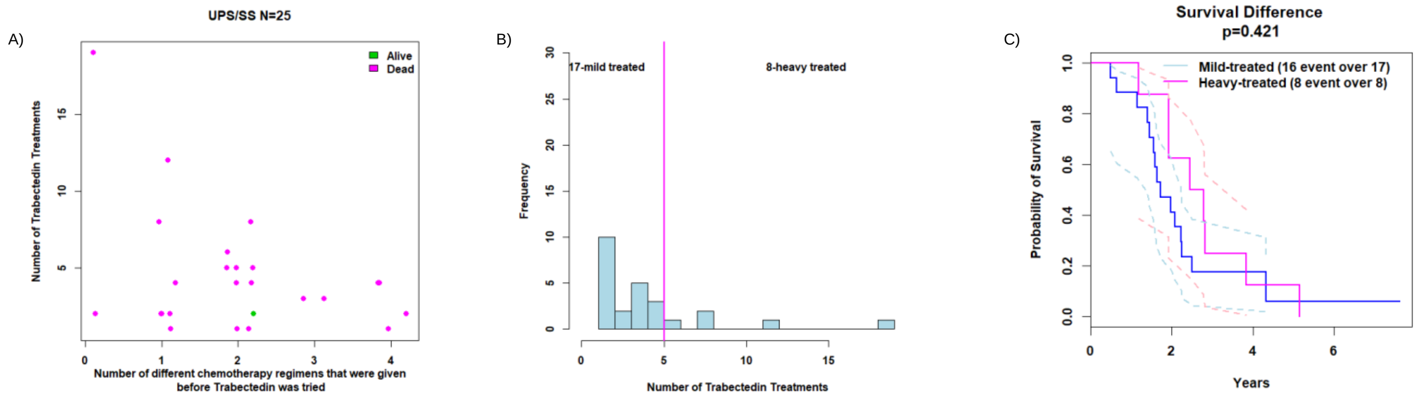

Figure S4. UPS/Spindle Cell Sarcoma subjects (*N* = 25). (**A**) Number of chemotherapy regimens prior to trabectedin therapy. (**B**) Patients with number of trabectedin treatments received. (**C**) Survival in relation to number of trabectedin treatments received, where heavy treated means greater than or equal to five treatments with trabectedin. No significant survival difference between groups.

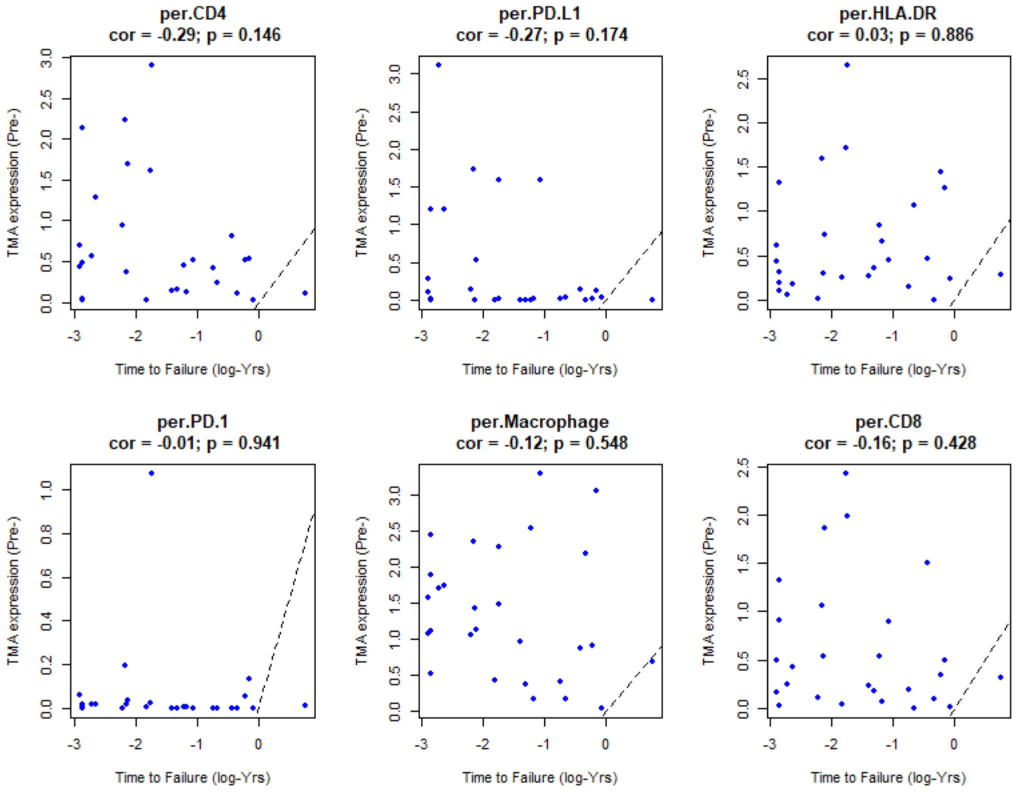

Figure S5. TMA expression versus time to trabectedin failure adjusted for tumor grade using Pearson Pairwise Correlation, correlation testing p values, and Benjamini-Hochberg adjustment (*q*-value) (Number of subjects *N* = 28).

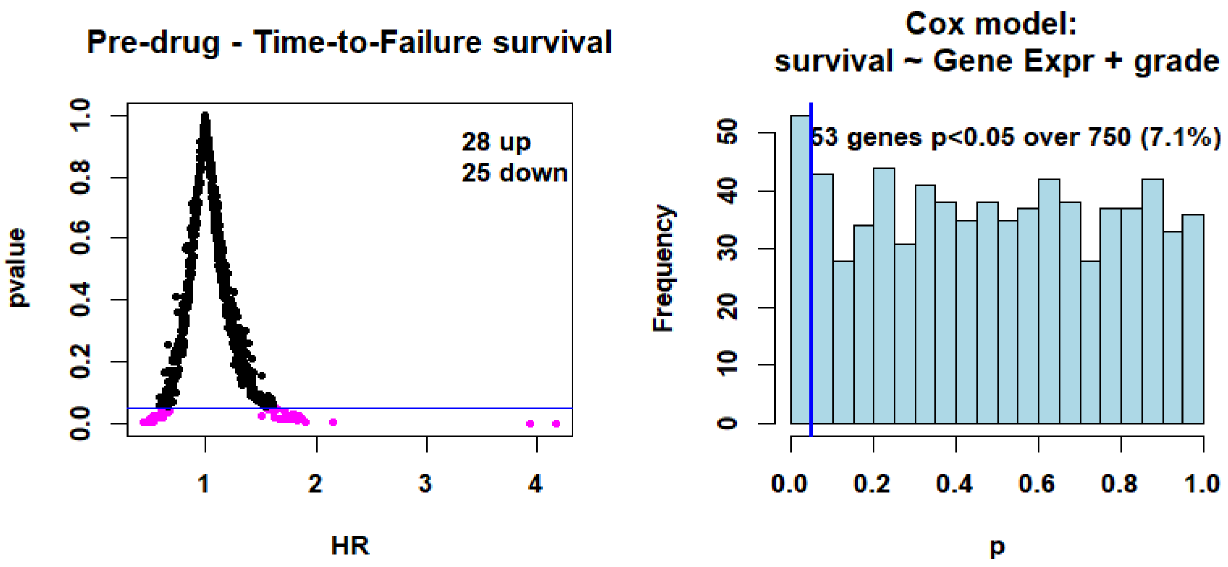

Figure S6. Cox regression to analyze pre-trabectedin treatment samples demonstrating that 53 genes expression data associated to time to trabectedin treatment failure: 28 genes with positive association, 25 genes with negative association. Tumor grade was adjusted for in this model.
